# Classification performance of sEMG and kinematic parameters for distinguishing between non-lame and induced lameness conditions in horses

**DOI:** 10.3389/fvets.2024.1358986

**Published:** 2024-04-02

**Authors:** Lindsay B. St. George, Tijn J. P. Spoormakers, Sarah Jane Hobbs, Hilary M. Clayton, Serge H. Roy, Jim Richards, Filipe M. Serra Bragança

**Affiliations:** ^1^Research Centre for Applied Sport, Physical Activity and Performance, University of Central Lancashire, Preston, United Kingdom; ^2^Department of Clinical Sciences, Equine Department, Faculty of Veterinary Medicine, Utrecht University, Utrecht, Netherlands; ^3^Department of Large Animal Clinical Sciences, College of Veterinary Medicine, Michigan State University, East Lansing, MI, United States; ^4^Delsys/Altec Inc., Natick, MA, United States; ^5^Allied Health Research Unit, University of Central Lancashire, Preston, United Kingdom

**Keywords:** equine, surface electromyography, gait analysis, ROC analysis, movement asymmetry, kinematics, sensitivity, specificity

## Abstract

Despite its proven research applications, it remains unknown whether surface electromyography (sEMG) can be used clinically to discriminate non-lame from lame conditions in horses. This study compared the classification performance of sEMG absolute value (sEMGabs) and asymmetry (sEMGasym) parameters, alongside validated kinematic upper-body asymmetry parameters, for distinguishing non-lame from induced fore- (iFL) and hindlimb (iHL) lameness. Bilateral sEMG and 3D-kinematic data were collected from clinically non-lame horses (*n* = 8) during in-hand trot. iFL and iHL (2–3/5 AAEP) were induced on separate days using a modified horseshoe, with baseline data initially collected each day. sEMG signals were DC-offset removed, high-pass filtered (40 Hz), and full-wave rectified. Normalized, average rectified value (ARV) was calculated for each muscle and stride (sEMGabs), with the difference between right and left-side ARV representing sEMGasym. Asymmetry parameters (MinDiff, MaxDiff, Hip Hike) were calculated from poll, withers, and pelvis vertical displacement. Receiver-operating-characteristic (ROC) and area under the curve (AUC) analysis determined the accuracy of each parameter for distinguishing baseline from iFL or iHL. Both sEMG parameters performed better for detecting iHL (0.97 ≥ AUC ≥ 0.48) compared to iFL (0.77 ≥ AUC ≥ 0.49). sEMGabs performed better (0.97 ≥ AUC ≥ 0.49) than sEMGasym (0.76 ≥ AUC ≥ 0.48) for detecting both iFL and iHL. Like previous studies, MinDiff Poll and Pelvis asymmetry parameters (MinDiff, MaxDiff, Hip Hike) demonstrated excellent discrimination for iFL and iHL, respectively (AUC > 0.95). Findings support future development of multivariate lameness-detection approaches that combine kinematics and sEMG. This may provide a more comprehensive approach to diagnosis, treatment, and monitoring of equine lameness, by measuring the underlying functional cause(s) at a neuromuscular level.

## 1 Introduction

Objective gait analysis and kinematic “upper-body” asymmetry parameters, expressed as differences between the minimum or maximum vertical displacement amplitudes of the horse’s head, withers, and pelvis (1), are increasingly used in equine veterinary practice to aid clinical decision making for lameness cases (2). The validity and clinical feasibility of kinematic asymmetry parameters for discriminating between non-lame and unilateral lameness conditions in horses has been demonstrated (1–9). These kinematic parameters provide primary, objective information about the degree and location of clinical or subclinical lameness, but the veterinarian must employ further clinical reasoning and evaluation (e.g., diagnostic imaging, analgesia) to determine the potential significance and aetiology of the measured/observed movement asymmetry (2, 10, 11). In humans, it is recognized that clinical gait analysis should go beyond kinematic measurements by studying the neuromuscular component of “resultant” movement, using surface electromyography (sEMG) (12–14). sEMG is the only non-invasive tool for quantifying isolated muscle activity and, when combined with kinematics, it provides a more comprehensive evaluation of a patient’s gait, as well as an empirical basis for identifying the functional cause(s) of gait abnormality (12–14). Scientific evidence supports the use of sEMG in human clinical gait analysis for improved diagnostics, as well as rehabilitation, treatment, and surgical planning and monitoring at a neurological level (12, 14–16). We propose that sEMG could similarly benefit the equine practitioner and researcher by measuring the pattern and degree of neuromuscular adaptations that contribute to, or are caused by, movement asymmetries. The combined use of sEMG and kinematic asymmetry parameters may provide more comprehensive processes for the objective diagnosis, treatment, and monitoring of equine lameness.

Recently, sEMG was used to conduct the first comparative study of adaptive muscle activity during induced, unilateral fore- (iFL) and hindlimb (iHL) lameness conditions (17, 18). When compared to the non-lame/baseline condition, significant bilateral changes in amplitude, quantified using the average rectified value (ARV), and the phasic activity pattern of sEMG signals from selected appendicular and axial muscles were observed during iFL and iHL (17, 18). However, despite its proven research applications and ability to detect muscular adaptations during equine lameness (17, 18), it remains unknown whether sEMG can be used clinically to classify non-lame and lame conditions in horses and, if so, what sEMG parameters can be recommended for making this distinction on the basis of sensitivity and specificity outcomes. This is critical information for developing sEMG as a potential tool to assist clinical decision-making. As a first step, evaluating and comparing the performance of selected sEMG parameters and kinematic asymmetry parameters for classifying non-lame and lame conditions, may provide informative, preliminary insights into whether sEMG can be used to accurately detect lameness at a neuromuscular level. Based on findings from the aforementioned studies (17, 18), we propose that ARV may offer a clinically feasible sEMG parameter for measuring the absolute activity within an individual muscle (sEMGabs) (17, 18). Further, in keeping with kinematic asymmetry parameters, ARV may also offer a measure of muscle asymmetry, when the difference in bilateral ARVs is calculated within a stride (sEMGasym). Thus, this study aims to determine whether selected sEMG parameters (sEMGabs and sEMGasym) from individual muscles can distinguish non-lame from iFL and iHL conditions in horses during overground trot, and to assess the classification performance of these sEMG parameters in relation to validated kinematic asymmetry parameters. We hypothesize that kinematic asymmetry parameters will generally exhibit better accuracy than sEMG parameters for detecting induced, unilateral lameness, but that certain sEMG parameters will exhibit comparable accuracy, depending on the bilateral or unilateral degree of adaptive changes in sEMG amplitude within an individual muscle during iFL or iHL.

## 2 Method

Ethical approval was obtained from Utrecht University (CCD: AVD108002015307) and the University of Central Lancashire (Reference number: RE/17/08a_b).

### 2.1 Horses

Eight (*n* = 8) horses (sex: 7 mares, one stallion, age: 9.2 ± 3.9 years, height: 161.3 ± 3.4 cm, body mass: 582.1 ± 39.4 kg, breed: 7 Dutch Warmblood, 1 Friesian) from the Utrecht University equine herd were used. Horses were in regular use for low-level dressage and pleasure riding. Horses were deemed as clinically non-lame (<1/5 AAEP Lameness Scale) through visual assessments by two qualified veterinarians (TS, FSB).

### 2.2 Instrumentation and equipment set up

To collect sEMG and three-dimensional (3D) kinematic data, horses were, respectively, instrumented with sEMG sensors (Delsys Trigno, Delsys Inc., United States) and retro-reflective markers (19 mm diameter super-spherical markers, Qualisys AB, Sweden). sEMG sensors were positioned to record bilaterally from the following superficial muscles: long head of triceps brachii (triceps), latissimus dorsi (latissimus), superficial gluteal (gluteal), vertebral head of biceps femoris (biceps), semitendinosus, and longissimus dorsi at the T14 (longissimus T14) and L1 (longissimus L1) vertebrae. Retro-reflective markers were attached over anatomical landmarks on the forelimbs, hindlimbs, head, and back. The reader is referred to St. George et al. (18) and Spoormakers et al. (17) for detailed descriptions of sEMG sensor and retro-reflective marker locations.

Overlying hair was clipped at each anatomical landmark and sEMG sensor site to ensure optimal adhesion and consistent placement across data collection sessions. Skin was then thoroughly cleaned using isopropyl alcohol. Saline solution was applied to each sEMG electrode and sensors were positioned over the muscle belly, with the electrodes oriented perpendicular to the underlying muscle fiber direction (19, 20), determined using ultrasonography. Sensors were attached to the skin using Delsys Adhesive Surface Interface strips (Delsys Inc., United States), combined with a drop of cyanoacrylate glue placed on top of double-sided tape, attached to the top and bottom of the sensor, above each electrode pair. Retro-reflective markers were attached using double-sided tape, with an additional drop of cyanoacrylate glue used to secure the hoof and distal limb markers.

An optical motion capture (OMC) system of eighteen high-speed, infrared cameras (Oqus 700+, Qualisys AB, Sweden) were used to collect 3D kinematic data. Cameras were secured to the walls of a large indoor hall at the Equine Department of Clinical Sciences of Utrecht University, where the study was conducted. The system was calibrated for each data collection session and produced an extended calibration volume approximately 56 m long and 10 m wide. The OMC system was hardware synchronized to the sEMG system to record both timeseries in one file.

### 2.3 Data acquisition protocol

sEMG (2000 Hz) and 3D kinematic (200 Hz) data were synchronously collected from each horse during in-hand trot trials, conducted on a straight, hard surfaced runway during control (baseline 1, baseline 2) and induced lameness (iFL, iHL) conditions. Data were collected using Qualisys Track Manager (Qualisys AB, Sweden) software, as the horse trotted over the runway four times, twice in each direction. One handler led the horses and permitted them to trot at their preferred velocity. Baseline 1 (non-lame) data were initially collected, then temporary, mild iFL (2–3/5 AAEP Lameness Scale) was induced by qualified veterinarians (TS, FSB) using a modified horseshoe lameness model to exert pressure on the sole of the hoof (21). The veterinarians graded and monitored the resulting lameness. In a cross-over design, horses were randomly divided into two groups (*n* = 4) for right and left iFL. Following iFL, trot trials were repeated. After a washout period of 24–48 h, the data collection process was repeated for baseline 2 and iHL conditions, which were randomized to the right (*n* = 4) or left (*n* = 4) HL. After each data collection session, the screw/sole pressure was removed. No horse showed adverse reactions to the mild, temporary lameness that was induced for this study.

### 2.4 Data analysis

Kinematic data were tracked in Qualisys Track Manager (Qualisys AB, Sweden) and imported into Visual3D (Version 2021.06.2, c-Motion Inc., United States) and Matlab (Version 2020b, TheMathWorks Inc., United States) software for further analysis. Gait event detection for stride segmentation and the calculation of kinematic asymmetry parameters (MinDiff and MaxDiff of poll, pelvis and withers, and Hip Hike during stance and swing phase) were conducted in Matlab. Gait events (hindlimb impact events) were detected in accordance with the method described by Roepstorff et al. (22) and kinematic asymmetry parameters were calculated in accordance with the methods described by Rhodin et al. (3) and Starke et al. (23). Asymmetry parameters were calculated for each stride using vertical displacement data from poll, withers and pelvis markers, which were high-pass filtered (Butterworth 4th order) with a cut-off frequency that was adjusted to the stride frequency of each measurement (24). Lameness induction was considered sufficient when the motion asymmetry difference between associated baseline and lameness induction conditions surpassed previously described reference values of 13 mm for head movement (MinDiff Poll or MaxDiff Poll) and 5 mm for pelvic motion asymmetry (MinDiff Pelvis and/or MaxDiff Pelvis) and with standard deviations less than their respective means (25).

Post-processing and analysis of sEMG signals was conducted in Visual3D and included DC-offset removal, high-pass filtering (Butterworth 4th order, 40 Hz cut-off) (26), and full-wave rectification. Gait events were manually imported into Visual3D for stride segmentation of sEMG data. The ARV was calculated using full-wave rectified signals with stride duration as the temporal domain. Outliers in ARV data were detected and removed by setting upper and lower outlier limits as two standard deviations outside the mean ARV values within each horse, muscle, and condition (27). Within-horse ARV data were normalized to the maximum value observed for each muscle across all strides from the corresponding baseline condition (28). The absolute, normalized ARVs from individual left- and right-side muscles within each stride represented the sEMGabs parameter. Ipsilateral hindlimb impact events were used for stride segmentation when calculating sEMGabs parameters, as left- and right-side muscles were analyzed separately (i.e., consecutive left hindlimb impact for left-side muscles and consecutive right hindlimb impact for right-hind muscles). Within each stride and muscle, sEMGasym was calculated by subtracting the normalized right-side ARV from the left-side ARV. In accordance with kinematic asymmetry parameters, left hindlimb impact events were employed for stride segmentation (3, 23) when calculating sEMGasym parameters to ensure that sEMG asymmetry was measured within the same temporal domain (i.e., consecutive left hindlimb impact for both left- and right-side muscles).

### 2.5 Statistical analysis

To increase statistical power, kinematic upper-body asymmetry parameters and sEMGasym parameters from right iFL and iHL were multiplied by −1 to mirror the indices and categorize all data as if they were derived from left lameness inductions only. sEMGabs parameters from right iFL and iHL were also mirrored. Therefore, results are reported from the “lame” side (LS) (ipsilateral to the side of induced lameness) and the “non-lame” side (NLS) (contralateral to the side of induced lameness). Receiver operating characteristic (ROC) curves were calculated in RStudio (Version 2023.0.1, RStudio, United States), using R package pROC (version 1.18.2) (29), for kinematic asymmetry and sEMG (sEMGabs, sEMGasym) parameters from baseline and the associated induced lameness conditions (baseline 1 and iFL, baseline 2 and iHL). Sensitivity, specificity, and cut-off values were calculated from the optimal values for each ROC curve using the Youden’s index. The ROC curves provide a graph of each measure’s performance for classifying non-lame and lame strides for each possible cut-off point, using sensitivity (the proportion of correctly classified lame strides) and specificity (the proportion of correctly classified “non-lame”/baseline strides) as the respective *y* and *x* axes. The closer the graph follows the left and top borders, the more accurate the test. Conversely, the closer the graph is to the diagonal, the less accurate the test. Area under the ROC curve (AUC) was calculated as a measure of “accuracy” for discriminating between baseline and induced lameness conditions. AUC values were interpreted as: excellent (AUC ≥ 0.90), good (0.9 > AUC ≥ 0.80), fair (0.80 > AUC ≥ 0.70), and poor (0.70 > AUC ≥ 0.60) discrimination, with 0.6 > AUC ≥ 0.50 indicating discrimination no better than chance (30, 31).

## 3 Results

A maximum of 254 strides were employed for the separate ROC analysis of sEMG parameters from the LS and NLS muscles from *n* = 8 horses during baseline and corresponding induced lameness conditions. A total of 647 strides were used for the separate ROC analysis of kinematic asymmetry parameters (163: baseline 1, 132: baseline 2, 189: iFL and 163: iHL). Descriptive statistics (mean ± SD) for the sEMG and kinematic asymmetry parameters, as well as stride speed and duration, are presented in Supplementary Table S1. The increase of MinDiff Poll and Pelvis confirmed a successful lameness induction (Supplementary Table S1), and this was consistent with the visual lameness assessment during iFL and iHL, respectively.

### 3.1 Classification performance of sEMG and kinematic parameters for induced forelimb lameness

**Figure 1:**
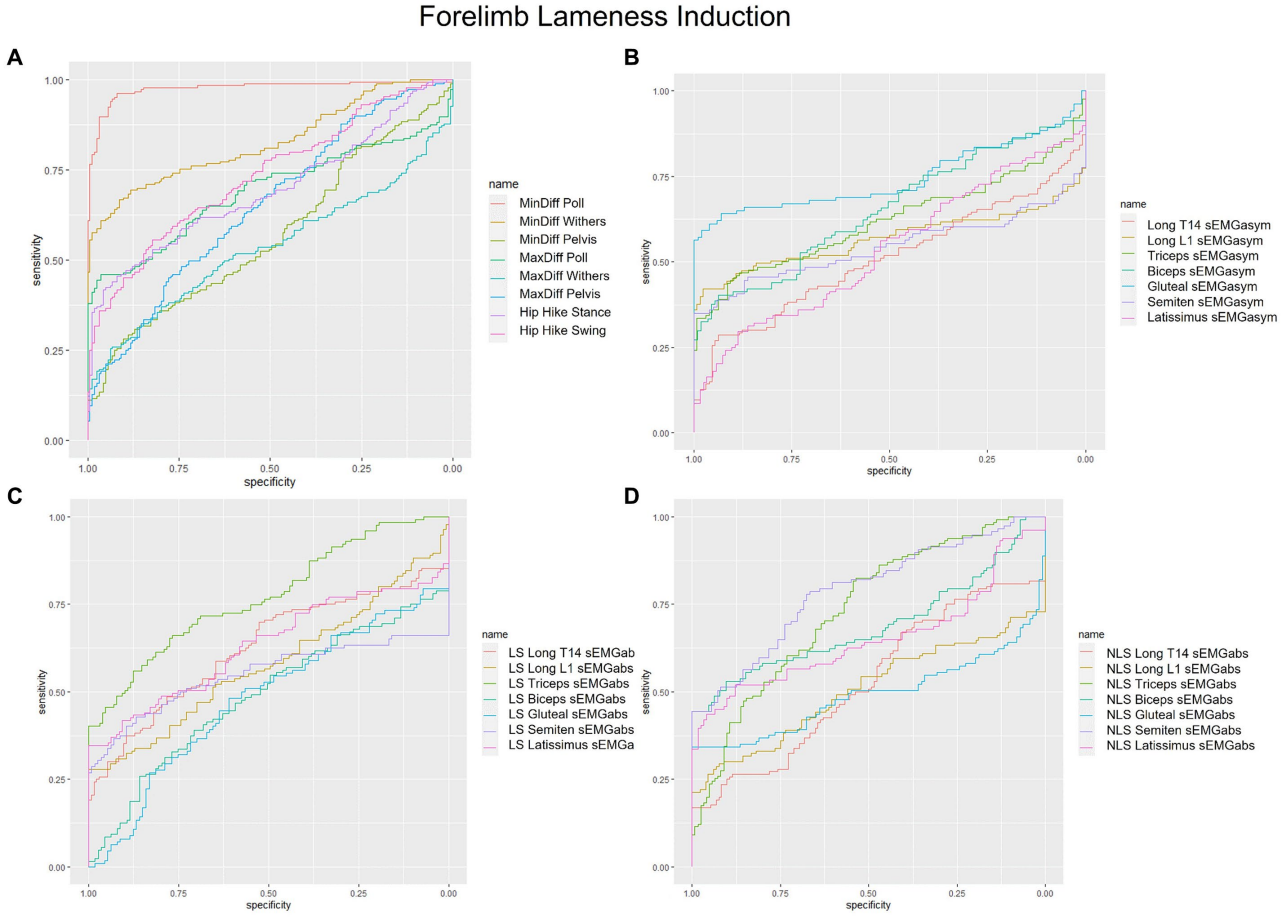
Receiver operating characteristic (ROC) curves for the detection of induced forelimb lameness based on **(A)** kinematic upper-body asymmetry parameters **(B)** sEMG asymmetry (sEMGasym) parameters for individual muscles **(C)** absolute sEMG (sEMGabs) parameters for inidivual lame side (LS) muscles, and **(D)** sEMGabs parameters for individual non-lame side (NLS) muscles.

sEMGabs parameters from LS and NLS triceps (AUC: 0.74 and 0.77, respectively), and NLS biceps and semitendinosus (AUC = 0.74) (Table 1 and Figures 1C,D), as well as gluteal and biceps sEMGasym parameters (AUC: 0.76 and 0.70, respectively) (Table 2 and Figure 1B) were the most accurate sEMG parameters for distinguishing iFL from non-lame conditions, exhibiting fair discrimination. All other sEMG parameters exhibited AUCs indicating poor to chance discrimination for iFL (AUC range: 0.49–0.69) (Tables 1, 2 and Figures 1B–D). MinDiff Poll showed excellent discrimination for iFL (AUC = 0.98), with all other kinematic asymmetry parameters ranging from fair to chance discrimination (AUC range: 0.53–0.79) (Table 2 and Figure 1A). A MinDiff cut-off score of −24.4 mm resulted in 97% sensitivity and 92% specificity for differentiating between iFL and non-lame conditions (Table 2).

**Table 1:** Receiver operating characteristic (ROC) analyses for discriminating between baseline (non-lame) and the associated induced fore- (iFL) or hindlimb (iHL) lameness trot strides, based on absolute sEMG parameters (sEMGabs) (%) from individual lame side (LS) and non-lame side (NLS) muscles from *n* = 8 horses.

Measure	Induction (iFL/iHL)	AUC	Sensitivity	Specificity	Cut-off Score
*sEMGabs*					
NLS Latissimus dorsi	iFL	0.63	0.49	0.92	76.22%
	iHL	0.68	0.61	0.79	91.02%
LS Latissimus dorsi	iFL	0.69	0.40	1.00	100.11%
	iHL	0.81	0.60	1.00	100.01%
NLS Triceps brachii	iFL	0.77	0.85	0.57	77.19%
	iHL	0.57	0.25	1.00	100.95%
LS Triceps brachii	iFL	0.74	0.56	0.86	91.68%
	iHL	0.62	0.42	1.00	100.10%
NLS Biceps femoris	iFL	0.74	0.50	1.00	100.30%
	iHL	0.97	0.91	1.00	100.28%
LS Biceps femoris	iFL	0.52	0.31	0.88	70.49%
	iHL	0.71	0.47	1.00	100.24%
NLS Superficial gluteal	iFL	0.55	0.38	1.00	121.44%
	iHL	0.73	0.59	1.00	100.21%
LS Superficial gluteal	iFL	0.49	0.49	0.65	81.47%
	iHL	0.90	0.73	1.00	100.57%
NLS Semitendinosus	iFL	0.74	0.46	1.00	103.15%
	iHL	0.72	0.51	1.00	108.36%
LS Semitendinosus	iFL	0.61	0.49	0.93	62.73%
	iHL	0.90	0.69	1.00	100.26%
NLS Longissimus dorsi T14	iFL	0.49	0.13	1.00	43.70%
	iHL	0.81	0.66	1.00	100.56%
LS Longissimus dorsi T14	iFL	0.59	0.39	0.88	76.90%
	iHL	0.91	0.82	0.87	96.38%
NLS Longissimus dorsi L1	iFL	0.51	0.28	0.93	73.80%
	iHL	0.67	0.35	1.00	100.41%
LS Longissimus dorsi L1	iFL	0.61	0.35	1.00	100.03%
	iHL	0.77	0.55	1.00	100.21%

**Table 2:** Receiver operating characteristic (ROC) analyses for discriminating between baseline (non-lame) and the associated induced fore- (iFL) or hindlimb (iHL) lameness trot strides, based on sEMG asymmetry measures (sEMGasym) (%) from individual muscles and kinematic upper-body asymmetry measures (mm) *n* = 8 horses.

Measure	Induction (iFL/iHL)	AUC	Sensitivity	Specificity	Cut-off Score
*sEMGasym*					
Latissimus dorsi	iFL	0.57	0.32	0.92	−10.62%
	iHL	0.62	0.63	0.67	−1.71%
Triceps brachii	iFL	0.62	0.47	0.89	−16.76%
	iHL	0.48	0.32	0.85	−14.66%
Biceps femoris	iFL	0.70	0.61	0.78	9.76%
	iHL	0.64	0.64	0.83	−16.16%
Superficial gluteal	iFL	0.76	0.62	0.97	22.61%
	iHL	0.59	0.50	0.83	12.22%
Semitendinosus	iFL	0.52	0.33	0.98	−29.44%
	iHL	0.76	0.60	1.00	−44.11%
Longissimus dorsi T14	iFL	0.51	0.28	0.92	−13.60%
	iHL	0.64	0.46	0.99	−15.35%
Longissimus dorsi L1	iFL	0.56	0.39	1.00	−15.44%
	iHL	0.61	0.38	0.90	−11.85%
*Upper-body asymmetry parameters*					
MinDiff Poll	iFL	0.98	0.97	0.92	−24.43 mm
	iHL	0.69	0.68	0.69	−11.03 mm
MinDiff Withers	iFL	0.79	0.61	0.90	−8.94 mm
	iHL	0.91	0.75	0.93	4.92 mm
MinDiff Pelvis	iFL	0.54	0.46	0.71	−2.47 mm
	iHL	0.96	0.90	0.93	−8.72 mm
MaxDiff Poll	iFL	0.77	0.56	1.00	−25.25 mm
	iHL	0.73	0.69	0.72	−9.21 mm
MaxDiff Withers	iFL	0.53	0.54	0.68	−6.64 mm
	iHL	0.86	0.89	0.69	−2.25 mm
MaxDiff Pelvis	iFL	0.67	0.49	0.76	11.65 mm
	iHL	0.99	0.99	0.94	−10.13 mm
Hip Hike Stance	iFL	0.66	0.47	0.90	21.09 mm
	iHL	0.99	0.99	0.96	−15.89 mm
Hip Hike Swing	iFL	0.71	0.68	0.65	12.32 mm
	iHL	1.00	0.96	1.00	−23.48 mm

### 3.2 Classification performance of sEMG and kinematic parameters for induced hindlimb lameness

**Figure 2:**
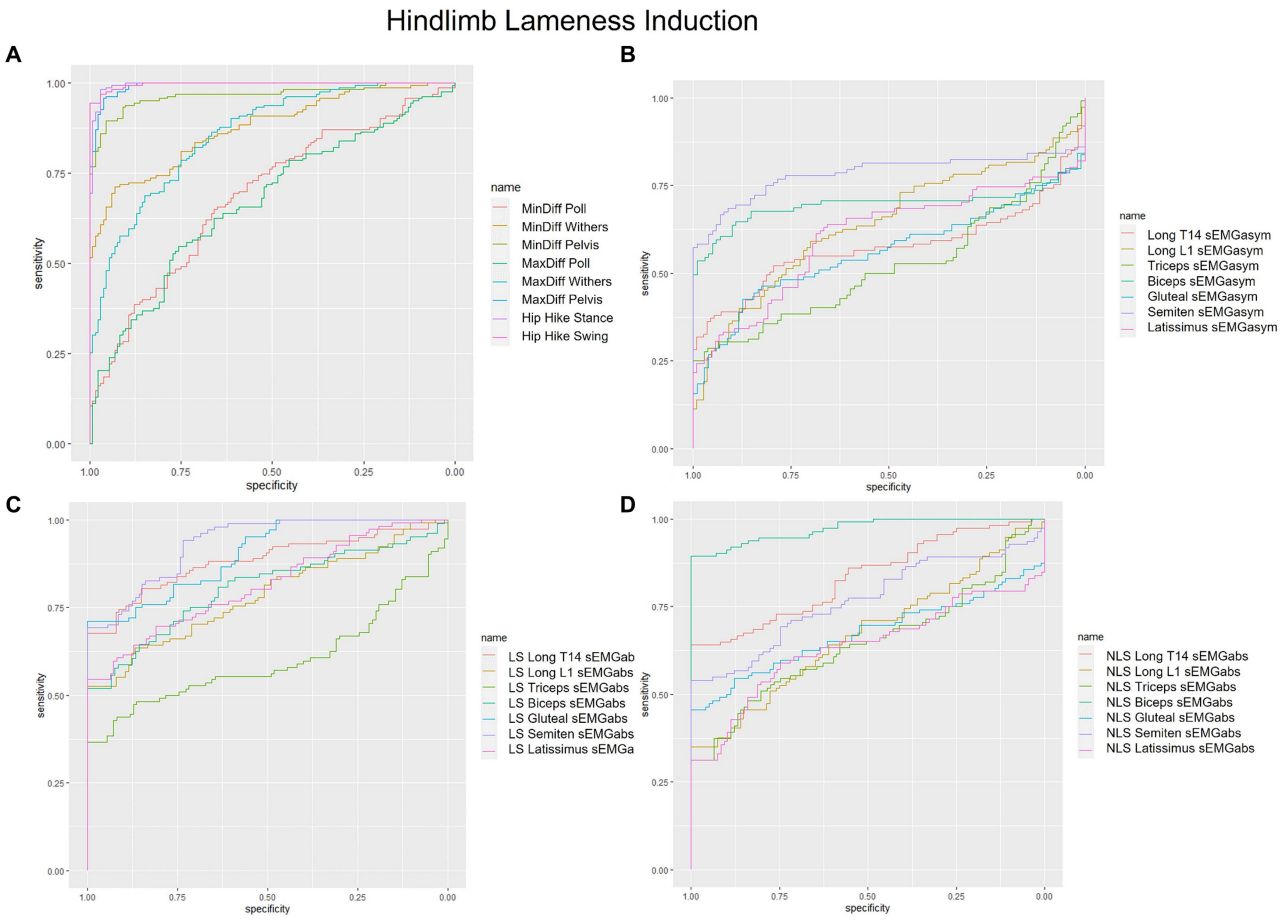
Receiver operating characteristic (ROC) curves for the detection of induced hindlimb lameness based on **(A)** kinematic upper-body asymmetry parameters **(B)** sEMG asymmetry (sEMGasym) parameters for individual muscles **(C)** absolute sEMG (sEMGabs) parameters for inidivual lame side (LS) muscles, and **(D)** sEMGabs parameters for individual non-lame side (NLS) muscles.

Non-lame side biceps sEMGabs was the most accurate (AUC = 0.97) sEMG parameter for distinguishing iHL from non-lame conditions (Table 1 and Figure 2D), followed by sEMGabs parameters from LS longissimus (T14), gluteal and semitendinosus, all of which exhibited an excellent discrimination (AUC range: 0.90–0.91) (Table 1 and Figure 2C). All other sEMGabs parameters exhibited good to chance discrimination (AUC range: 0.57–0.81) for iHL (Table 1 and Figures 2C,D). Cut-off scores of 100.3–100.5% for NLS biceps, LS semitendinosus, and LS gluteal sEMGabs parameters resulted in 100% specificity and >69% sensitivity (Table 1). The semitendinosus sEMGasym parameter exhibited acceptable accuracy (AUC = 0.76) for differentiating iHL from non-lame conditions, but all other sEMGasym parameters showed poor to chance discrimination (AUC range: 0.48–0.64) (Table 2 and Figure 2B). MinDiff and MaxDiff Pelvis, MinDiff Withers, and Hip Hike parameters showed excellent discrimination for iHL (AUC range: 0.91–0.99), with all other parameters ranging from good to poor discrimination (AUC range: 0.68–0.86) (Table 2 and Figure 2A).

## 4 Discussion

In this study, ROC analysis was used to measure and compare the classification performance of two amplitude-based sEMG parameters (sEMGabs, sEMGasym) from individual muscles for distinguishing between non-lame and iFL and iHL conditions during in-hand trot. In addition, kinematic upper-body asymmetry parameters were evaluated in the same way, so that the classification performance of sEMG parameters could be compared against validated outcome measures for lameness detection (1–9, 32). sEMGabs performed better than sEMGasym for detecting both iFL and iHL across all studied muscles. This finding suggests that evaluating changes in sEMG amplitude from individual left- and right-side muscles (sEMGabs) may be more meaningful than evaluating bilateral changes within a muscle (sEMGasym). Kinematic findings largely agreed with the literature and supported our hypothesis, with head and pelvis asymmetry parameters exhibiting the best accuracy for detecting iFL and iHL, respectively (1, 2, 9). Overall, sEMG parameters had a better ability to detect iHL than iFL, with some sEMGabs parameters exhibiting comparable or better accuracy than established pelvic asymmetry parameters. Thus, sEMG findings supported our hypothesis, as the best-performing sEMG parameter(s) varied across individual muscles and induced lameness conditions, with some exhibiting comparable accuracy to kinematic asymmetry parameters.

Several sEMGabs parameters displayed excellent discriminative ability for detecting iHL. Of these, NLS biceps sEMGabs was the best sEMG parameter for detecting iHL with AUC comparable to MinDiff Pelvis, which is described as the optimal parameter for distinguishing horses with hindlimb impact-type lameness (33, 34). Indeed, significant bilateral increases in biceps ARV have been observed during iHL and these increases were comparatively greater for the NLS biceps than LS biceps, which was consistently observed across horses (18). All other sEMGabs parameters with excellent discriminative ability (AUC > 0.90) for iHL were LS muscles, specifically gluteal, semitendinosus, and longissimus T14. Significant bilateral increases in ARV have also been observed for these muscles during iHL, but in contrast to the biceps, these increases were comparatively greater on the LS than the NLS (17, 18). It has been hypothesized that the biceps plays a larger role in stabilizing the more vertically loaded NLS hindlimb and/or the generation of greater propulsion observed in this limb during iHL (35–37). In contrast, greater sEMGabs increases of the LS semitendinosus and gluteal may reflect increased requirements of these muscles for mitigating loading in the affected LS hindlimb (18). In longissimus, more pronounced ARV increases have been observed at the LS T14 location than at the NLS location during thoracolumbar extension, possibly reflecting increased requirements for stabilizing the trunk against compensatory sagittal plane forces during iHL (1, 17). Further studies are required to confirm these observations and theories, especially as horses exhibited individual variation in their response to iHL, particularly in the LS muscles. Thus, within the context of this study, the changes observed in NLS biceps sEMGabs appear to be the best indicator of iHL, but further work is required to confirm these preliminary findings.

In contrast to iHL, no sEMG parameter exhibited better than fair discriminative power for detecting iFL, with the majority exhibiting poor or chance discrimination. The generally poorer performance of sEMG parameters for detecting iFL may be related to the fact that compensatory changes in sEMG amplitude during iFL were generally more variable across horses in comparison to those observed during iHL, particularly for the parameters mentioned in the above paragraph (17, 18). NLS triceps sEMGabs was the best sEMG parameter for detecting iFL, with NLS biceps, NLS semitendinosus, and LS triceps sEMGabs parameters exhibiting similar AUC values. Indeed, significant bilateral increases for biceps and semitendinosus sEMGabs have been observed during iFL, but these were comparatively higher in the NLS, reflecting increased muscular requirements to stabilize this hindlimb, which undergoes increased compensatory loading within the lame diagonal pair (18, 37–39). Opposing bilateral adaptations have been observed for the triceps during iFL, with significant decreases in sEMGabs on the NLS and significant increases on the LS, reflecting muscular compensations to mitigate increased vertical impulse on the more loaded NLS forelimb, and to damp vertical forces on the affected lame forelimb (18, 37–39). Given these opposing bilateral changes in triceps sEMGabs, one might expect that the triceps sEMGasym parameter would perform well for distinguishing iFL, but this parameter exhibited poor discriminative ability (AUC = 0.62). In fact, all sEMGasym parameters exhibited low sensitivity and specificity for detecting both iFL and iHL, with no parameter displaying anything better than fair discriminative power. This can be explained by significant bilateral increases in sEMG amplitude that were observed across most muscles studied here during iFL and iHL (17, 18), which serve to “cancel out” measured changes that occur in response to induced lameness. As such, findings from this study demonstrate that measuring the behavior of individual muscles, or sEMGabs parameters, results in superior performance for distinguishing iFL and iHL from non-lame conditions, when compared to sEMGasym parameters.

To our knowledge, this is the first study to use an induced lameness model to evaluate the accuracy of kinematic asymmetry parameters for differentiating between non-lame and lame conditions. Across all studied parameters, MinDiff Poll and Hip Hike (stance and swing) were the most accurate for distinguishing iFL and iHL, respectively. For iHL, MinDiff and MaxDiff Pelvis and MinDiff Withers had comparable AUCs (>0.90) to the Hip Hike parameter, which indicated excellent discriminative power for iHL. Two recent studies have employed ROC analysis to evaluate the accuracy and associated threshold values of kinematic asymmetry parameters, measured using IMUs (33, 40), for differentiating between non-lame and lame groups of horses, as defined using visual lameness evaluation by expert veterinarians (33, 40). Our general findings agree with these studies, in that pelvic asymmetry parameters had a higher discriminative power (as quantified by higher AUC values) for detecting iHL than head and withers asymmetry parameters for detecting iFL (33, 40).

Although the purpose of this study was not to define threshold values for kinematic asymmetry parameters, it is interesting to note that the cut-off scores reported here for MinDiff and MaxDiff Pelvis are similar (within 1.2 mm) to those reported by Pfau et al. (33), who used analogous kinematic parameters to measure upper-body movement asymmetry in a group of thoroughbred racehorses. Here, these cut-off scores resulted in sensitivity and specificity values ≥90%, with the same thresholds showing specificity values >80% and sensitivity values of 90 and 50% for MinDiff and MaxDiff Pelvis, respectively, in the study by Pfau et al. (33). Thus, findings from this study, which are based on standardised, induced lameness, may support the thresholds of Pfau et al. (33), which were aligned with visually identified lameness. In contrast, we observed an absolute cut-off score of 24 mm for MinDiff Poll, which is higher than the 14.5 mm threshold reported by Pfau et al. (33) for the same parameter, but with comparatively higher sensitivity and specificity values (>90%) observed here. These differences could be explained by several methodological differences between studies, but may also be linked to the comparatively higher variation that has been reported for head asymmetry parameters than for pelvis parameters (25, 41). These findings further support the notion to define breed-, age-, and discipline/use-specific asymmetry thresholds for equine gait analysis (33, 42). Still, our overall kinematic findings agree with the literature, which describes head and pelvic asymmetry parameters as the most sensitive indicators of fore- and hindlimb lameness for both subjective and objective lameness evaluation (1, 2, 33, 40).

There are some limitations that should be considered when evaluating the results of this preliminary study. This study was limited to the evaluation of two amplitude-based sEMG parameters, calculated from selected superficial muscles in a relatively small sample of horses, which can be considered limitations. Using an acute induced lameness model provided a known diagnosis that was standardized across subjects, which may explain the generally higher AUC values observed here for kinematic asymmetry parameters compared to studies that have employed subjective diagnosis of naturally occurring lameness (33, 40). Further, the lameness induction model offered a unique opportunity to study the proportional change in muscle activity between baseline and induced lameness conditions by normalizing sEMG parameters to each horse’s baseline condition. Therefore, several sEMG parameters exhibit cut-off scores >100%. However, normalization to a baseline condition is unlikely to be clinically feasible, as veterinarians generally do not have access to the non-lame reference data of a horse that is presented for lameness examination. This can be considered another limitation of the study, and thus, further research is required to develop “stand-alone” sEMG parameters and associated cut-off scores that constitute clinically meaningful and sensitive neuromuscular changes in a larger cohort of clinical lameness cases. Importantly, further research is required to determine normative sEMG profiles for non-pathological equine gait, that can be used as a reference for developing such “stand alone” sEMG parameters (43). Future research should also aim to uncover which muscles are best for detecting equine lameness and this information could be used to streamline data acquisition processes for future clinical applications. Finally, it is important to note that this study was not intended as a classification performance “contest” between sEMG and kinematic parameters for distinguishing lameness conditions, but rather to validate discriminant ability from different, albeit related domains. The next step, which was beyond the scope of this study, is to employ multivariate analysis to evaluate how kinematic and sEMG parameters can be optimally combined to distinguish lameness conditions in horses.

Findings from this study suggest that, across all studied muscles, sEMGabs parameters exhibit better accuracy than sEMGasym parameters for distinguishing both iFL and iHL from non-lame conditions within an individual horse. Further, sEMG parameters performed better for distinguishing iHL from non-lame conditions than for detecting iFL. This is an encouraging finding, particularly given that hindlimb lameness is generally more difficult for veterinarians to detect than forelimb lameness (44, 45). sEMG appears to be a promising tool to aid clinical decision making, particularly given the comparable AUCs between established kinematic asymmetry parameters and some of the sEMG parameters evaluated here. This suggests that sEMG could be considered as a useful adjunct to established kinematic asymmetry parameters, as it provides a unique perspective on underlying muscle activation and control when trying to clinically interpret the movement asymmetry observed. Thus, with further development, sEMG may offer a complementary addition to a veterinarian’s diagnostic toolkit, particularly for mild/moderate or complex lameness cases. Findings from this preliminary study justify further work to develop and assess additional sEMG parameters that are stand-alone, sensitive, clinically meaningful, and can be combined with kinematic asymmetry parameters to optimally distinguish between normal and abnormal equine gait.

## Data availability statement

The raw data supporting the conclusions of this article will be made available by the authors, without undue reservation.

## Ethics statement

The animal study was approved by Utrecht University (CCD: AVD108002015307) and the University of Central Lancashire (Reference number: RE/17/08a_b). The study was conducted in accordance with the local legislation and institutional requirements.

## Author contributions

LS: Conceptualization, Data curation, Formal analysis, Funding acquisition, Investigation, Methodology, Project administration, Resources, Software, Validation, Visualization, Writing – original draft, Writing – review & editing. TS: Conceptualization, Data curation, Investigation, Methodology, Project administration, Resources, Validation, Writing – review & editing, Supervision. SH: Conceptualization, Methodology, Resources, Validation, Writing – review & editing, Supervision. HC: Conceptualization, Methodology, Validation, Writing – review & editing, Supervision. SR: Conceptualization, Methodology, Supervision, Validation, Writing – review & editing. JR: Conceptualization, Methodology, Supervision, Validation, Writing – review & editing. FS: Conceptualization, Data curation, Formal analysis, Investigation, Methodology, Project administration, Resources, Software, Supervision, Validation, Visualization, Writing – review & editing.
