# Supplementary material for: Classification performance of sEMG and kinematic parameters for distinguishing between non-lame and induced lameness conditions in horses
Source: Front Vet Sci. 2024 Apr 2;11:1358986. doi: 10.3389/fvets.2024.1358986 (PMC11018915; doi:10.3389/fvets.2024.1358986)
Supplement: Supplementary file 1 [file Table_1.docx]

Table S1. Descriptive statistics (mean ± standard deviation) for kinematic upper-body asymmetry parameters and sEMG parameters (sEMGabs, sEMGasym) from individual muscles from n = 8 horses. LS: lame side, NLS: non-lame side, iFL: induced forelimb lameness, iHL: induced hindlimb lameness.

| **Measure** | **Condition** | | | |
| --- | --- | --- | --- | --- |
|  | **Baseline 1** | **iFL** | **Baseline 2** | **iHL** |
| Stride Speed (m/s) | 3.10 ± 0.27 | 2.98 ± 0.26 | 3.04 ± 0.38 | 2.97 ± 0.37 |
| Stride Duration (s) | 0.74 ± 0.03 | 0.74 ± 0.03 | 0.75 ± 0.03 | 0.74 ± 0.03 |
| **Upper-body asymmetry parameters** | | | | |
| MinDiff Poll (mm) | -1.37 ± 15.94 | -58.63 ± 28.70 | 16.93 ± 38.80 | -15.44 ± 21.17 |
| MinDiff Withers (mm) | 0.54 ± 6.68 | -16.59 ± 10.55 | -1.62 ± 5.39 | 11.43 ± 9.87 |
| MinDiff Pelvis (mm) | -2.21 ± 6.67 | 2.95 ± 8.92 | -2.11 ± 5.74 | -21.54 ± 11.04 |
| MaxDiff Poll (mm) | -5.52 ± 14.05 | -27.29 ± 32.32 | -3.53 ± 18.17 | -12.87 ± 16.79 |
| MaxDiff Withers (mm) | -0.62 ± 7.04 | -6.75 ± 10.12 | -0.23 ± 8.62 | 4.82 ± 7.48 |
| MaxDiff Pelvis (mm) | -2.22 ± 9.96 | 9.40 ± 10.13 | 4.91 ± 8.09 | -26.43 ± 13.57 |
| Hip Hike Stance (mm) | -3.63 ± 12.90 | 20.89 ± 16.15 | 1.11 ± 14.09 | -47.92 ± 23.03 |
| Hip Hike Swing (mm) | -4.73 ± 13.26 | 19.03 ± 16.28 | 2.34 ± 11.98 | -57.36 ± 25.48 |
| **sEMGasym** | | | | |
| Latissimus dorsi (%) | -2.87 ± 12.52 | -14.07 ± 28.28 | -1.03 ± 11.17 | -0.81 ± 51.54 |
| Triceps brachii (%) | 3.69 ± 18.38 | -29.55 ± 25.96 | 1.28 ± 15.22 | 37.18 ± 107.57 |
| Biceps femoris (%) | 4.32 ± 14.82 | 29.05 ± 56.47 | 3.80 ± 16.45 | 78.75 ± 153.41 |
| Superficial gluteal (%) | -2.24 ± 12.40 | 24.91 ± 118.91 | -3.24 ± 14.56 | -26.07 ± 47.62 |
| Semitendinosus (%) | 4.41 ± 15.28 | 29.57 ± 92.62 | -0.56 ± 17.14 | -45.83 ± 191.16 |
| Longissimus dorsi T14 (%) | -1.13 ± 9.84 | -5.65 ± 19.60 | -1.87 ± 11.76 | -6.33 ± 34.33 |
| Longissimus dorsi L1 (%) | -1.15 ± 8.14 | -85.24 ± 151.10 | -0.28 ± 9.62 | 7.08 ± 23.05 |
| **sEMGabs** | | | | |
| NLS Latissimus dorsi (%) | 81.39 ± 10.08 | 74.48 ± 16.80 | 80.91 ± 10.24 | 100.65 ± 55.21 |
| LS Latissimus dorsi (%) | 82.83 ± 9.53 | 89.07 ± 26.22 | 83.13 ± 8.99 | 102.41 ± 19.18 |
| NLS Triceps brachii (%) | 77.20 ± 11.46 | 67.35 ± 11.87 | 82.58 ± 11.43 | 127.44 ± 105.39 |
| LS Triceps brachii (%) | 77.38 ± 11.56 | 97.54 ± 22.99 | 79.44 ± 14.28 | 90.79 ± 25.04 |
| NLS Biceps femoris (%) | 81.59 ± 11.58 | 111.05 ± 53.06 | 80.07 ± 12.94 | 187.87 ± 124.36 |
| LS Biceps femoris (%) | 78.37 ± 12.89 | 84.25 ± 22.51 | 75.95 ± 15.27 | 116.60 ± 60.25 |
| NLS Superficial gluteal (%) | 82.56 ± 9.38 | 130.75 ± 88.66 | 80.74 ± 11.56 | 94.94 ± 25.34 |
| LS Superficial gluteal (%) | 84.77 ± 9.25 | 106.15 ± 57.90 | 87.13 ± 8.92 | 121.45 ± 34.38 |
| NLS Semitendinosus (%) | 76.32 ± 12.39 | 171.82 ± 186.29 | 75.30 ± 13.20 | 129.18 ± 58.75 |
| LS Semitendinosus (%) | 74.29 ± 15.44 | 95.54 ± 57.66 | 76.49 ± 12.56 | 154.94 ± 139.34 |
| NLS Longissimus dorsi T14 (%) | 85.16 ± 9.97 | 77.64 ± 29.54 | 83.84 ± 11.04 | 109.19 ± 26.73 |
| LS Longissimus dorsi T14 (%) | 84.42 ± 9.14 | 80.94 ± 19.67 | 86.04 ± 9.51 | 116.59 ± 26.30 |
| NLS Longissimus dorsi L1 (%) | 84.64 ± 8.53 | 77.71 ± 22.11 | 84.88 ± 10.45 | 101.98 ± 22.38 |
| LS Longissimus dorsi L1 (%) | 85.57 ± 8.51 | 159.73 ± 155.77 | 85.00 ± 10.19 | 95.27 ± 15.06 |
